# Does the Sound of a Singing Bowl Synchronize Meditational Brainwaves in the Listeners?

**DOI:** 10.3390/ijerph20126180

**Published:** 2023-06-19

**Authors:** Seong-Chan Kim, Min-Joo Choi

**Affiliations:** 1 Interdisciplinary Postgraduate Program in Biomedical Engineering, Jeju National University, 102 Jejudaehak-ro, Jeju-si 63243, Republic of Korea; 2 Department of Medicine, College of Medicine, Jeju National University, 102 Jejudaehak-ro, Jeju-si 63243, Republic of Korea

**Keywords:** singing bowl, beat frequency, brain wave, meditation, synchronization, activation

## Abstract

This study aims to verify if the beating sound of a singing bowl synchronizes and activates brain waves during listening. The singing bowl used in this experiment produce beats at a frequency of 6.68 Hz, while it decays exponentially and lasts for about 50 s. Brain waves were measured for 5 min in the F3 and F4 regions of seventeen participants (eight males and nine females, average age 25.2) who heard the beating singing bowl sounds. The experimental results showed that the increases (up to ~251%) in the spectral magnitudes of the brain waves were dominant at the beat frequency compared to those of any other clinical brain wave frequency bands. The observed synchronized activation of the brain waves at the beating sound frequency supports that the singing bowl sound may effectively facilitate meditation and relaxation, considering that the beat frequency belongs to the theta wave region which increases in the relaxed meditation state.

## 1. Introduction

A singing bowl is a bowl-shaped percussion instrument [1]. The singing bowl has a peculiar feature in that it sounds as well as creates a beat, lasting for a long time after it has been played [2]. The singing bowl sound has often been used to reduce the degree of tension, anxiety, and depression [3]. The singing bowl sound is known to facilitate physiological and psychological responses, such as stabilizing blood pressure and heart rate [2,4]. Although the singing bowl sound is reported to give positive effects in meditation or alternative medicine, the mechanism of its psychoacoustic effects remains unclear [3,4].

It is presumed that the singing bowl sound may play a critical role in the beneficial responses of the brain through its strong beat. If the brain waves are activated and synchronized at the beat frequencies located in theta waves, the brain is likely shifted to a relaxed meditation state [1]. Meditation effects that evoke psychophysiological changes may result in increases in the theta waves [5,6,7,8,9]. However, no systematic study on such synchronized activation has been reported. The present study aims to examine if the singing bowl beating sound gives rise to a significant increase in the brain waves (electroencephalogram, EEG) being dominant at the beating frequency.

## 2. Materials and Methods

A total of seventeen participants (male: 8, female: 9, average age: 25.2 ± 3.5) participated in this study. They were healthy adults without hearing disabilities, cognitive difficulties or neurological damage. Participants were voluntarily recruited from the University in Jeju, Korea. EEG was performed on participants who voluntarily consented after hearing an explanation about the purpose of the study, the experimental method, the right to voluntarily participate in the study as a research subject, and the right to withdraw consent to participate. The experiment was conducted under the approval of Jeju National University Hospital Institutional Review Board (IRB) (JEJUNUH 2018-10-010).

**Figure 1:**
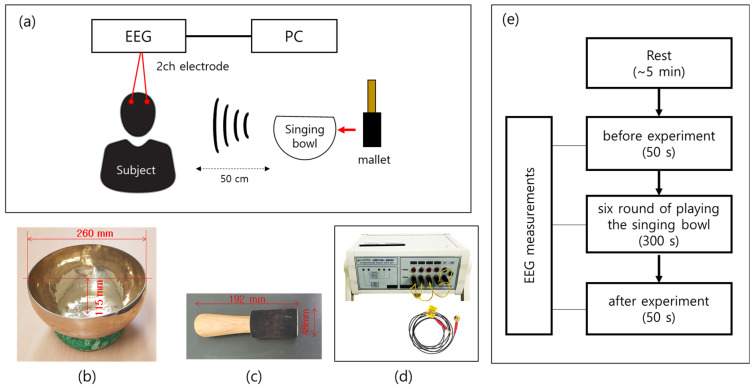
Experimental setup and method: (**a**) schematic illustration of the experimental space and method, (**b**) singing bowl, (**c**) mallet, (**d**) EEG measurement device with a wet electrode and (**e**) experimental procedure.

In this experiment, the changes in the spectral magnitude of the brain waves were monitored while the participants heard the singing bowl sound. The singing bowl sound was recorded to measure its beating spectral property. The experimental setup, tools and procedure are shown in Figure 1.

### 2.1. Singing Bowl Sound

The singing bowl used in this study is 260 mm in diameter and 115 mm in depth, a product of Best Himalaya, Nepal (Figure 1a,b). It was played with a cylindrical mallet of 192 mm in height and 48 mm in diameter (Figure 1c). Each percussion produces a sound modulated with a strong beat that lasts for about 50 s. Figure 1a illustrates a schematic overview of the experimental tools and space, including the relative location between the singing bowl and the subject.

### 2.2. Acoustic Apparatus for Recording and Acoustic Analysis

The singing bowl sound was recorded using a mobile sound analysis system (NoiseBook, 4820MHS II, Head Acoustics). The frequency characteristics of the recorded sound were analyzed using FFT in MATLAB. In order to determine the spectral properties of the low frequency beating phenomenon of the singing bowl sound, we first reconstructed the envelope of the recorded sound signal using a Hilbert transform. The frequency spectrum of the envelope was then plotted in the frequency range of 0~50 Hz employed in clinical EEG.

### 2.3. Brain Wave Measurements

Brain waves were recorded at the F3 and F4 positions of the international standard 10–20 system on the left and right sides of the dorsolateral prefrontal cortex (DLPFC), known to be sensitive to brain activity during meditation [5,8,10,11,12,13,14]. The EEG signals were acquired using an EEG measurement instrument (LXE1104, Laxtha, Republic Korea) via wet electrodes (Figure 1d). The measured EEG signals were stored on a PC in digital form with the sampling rate of 256 Hz. The participants in this study comprised 17 healthy adults with normal hearing, as confirmed by a hearing test conducted using an Audiometer (120 Audiometer, Beltone, Chicago, IL, USA). In addition, verbal confirmation was received to ensure that the participants had no history of any auditory disorders or diseases.

Figure 1e presents a flow chart of the entire experiment which took about 700 s. The participants laid down on a comfortable bed-chair. After the electrodes were attached, they closed their eyes for approximately 5 min in a relaxed position. When a stable EEG was observed, the EEG was recorded for 50 s. After that, the singing bowl was played 6 times for 5 min at intervals of 50 s, and at the same time, the brain waves were recorded. After the sixth round of playing the percussion instrument, an additional EEG was measured for 50 s without listening to the singing bowl sound. All experiments were conducted with the participants who had their eyes closed.

### 2.4. EEG Analysis

The measured time history of the brain waves was converted into the spectral magnitude or power of each clinical frequency band of EEG via FFT. The clinical frequency bands are divided into the five spectral regions: delta (0~4 Hz), theta (4~8 Hz), alpha (8~13 Hz), beta (13~30 Hz) and gamma (30~50 Hz). The spectral powers of the brain waves were compared before and after listening to the singing bowl sound to examine changes in the brain waves of the participants. In order to test the temporal response of the brain waves to the singing bowl sound, temporal variations in the changes in magnitude of each spectral band of EEG were monitored at a time interval of 50 s. The spectral band powers of each subject were normalized to the total spectral power (0~50 Hz) to eliminate the variability in the degree of subject-to-subject EEG activity.

## 3. Results

**Figure 2:**
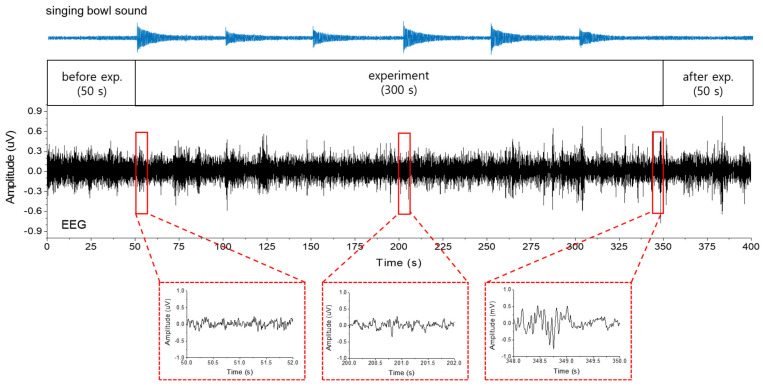
The temporal history of the repeating singing bowl sound (top) and the brain wave (middle) of the subject listening to the singing bowl sound. The bottom three panels magnify the brain waves in the time axis, recorded at the beginning, in the middle and at the end of the experiment.

The measured time history of the singing bowl sounds (top) and brain waves (middle) are presented in Figure 2. The three bottom panels magnify the brain waves recorded at the characteristic temporal locations (beginning, halfway and end) of the experiment, illustrating that the magnitudes of the brain waves increase with time and are significantly larger at the end of the experiment than those at the beginning. The increase was apparent in the low frequency components, as seen in the magnified figures. These types of changes in EEG are known to be common in psychological relaxation or meditation [5].

### 3.1. Temporal and Spectral Characteristics of the Singing Bowl Sound

**Figure 3:**
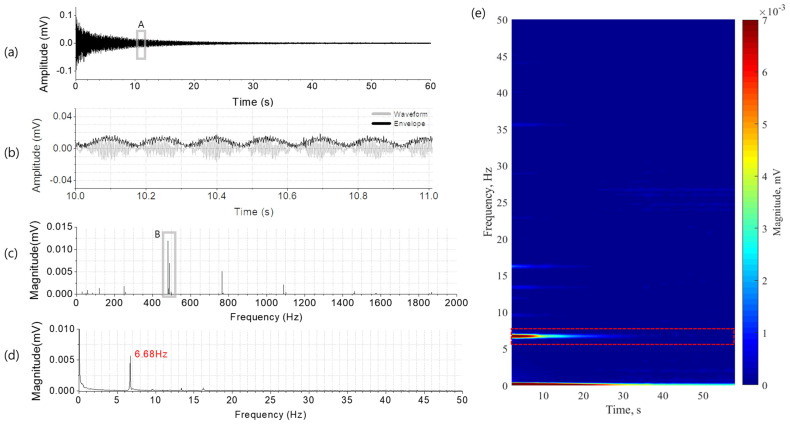
A typical singing bowl sound plotted in the time and frequency domain: (**a**) the waveform, (**b**) a part of the waveform in box A magnified to clearly display the envelope, (**c**) the frequency spectrum of the entire singing bowl sound waveform, (**d**) the frequency spectrum of the envelope and (**e**) the time–frequency representation of the envelope calculated with a window length of 4 s at a time interval of 0.5 s.

Figure 3 shows a typical measured waveform of the singing bowl sound. It gradually diminishes in amplitude for more than 40 s after hitting the percussion instrument and persists for approximately 50 s (Figure 3a). A part of the waveform (marked by ‘A’) was expanded in the time axis to reveal a low frequency variation of the sound, which is called a beat (Figure 3b). It was observed that the beat repeated at an interval of approximately 0.15 s.

Figure 3c is the frequency spectrum of the singing bowl sound. The fundamental frequency (marked ‘B’) that determines the pitch of the singing bowl sound was found to be 482.61 Hz. This frequency corresponds to a B4 note in the musical scale. As seen in Figure 3c, the singing bowl sound contains not only the fundamental frequency but also additional spectral components. The spectral components were observed at 773.15 Hz, 1102.56 Hz, 1464.81 Hz and 1870.86 Hz, corresponding to the musical scales near G5, C#6, F#6 and A#6, respectively. The number and magnitude of these spectral components determine the tonal property of the singing bowl sound. In addition, as seen in box ‘B’ in Figure 3c, an additional frequency component (relatively small but significant) appears near the fundamental frequency (482.61 Hz). The minute frequency difference of 6.68 Hz between them causes the beating phenomenon.

In order to calculate the frequency spectrum of the beat, we reconstructed its time domain signal using a Hilbert transform, plotted in Figure 3b as the envelope of the singing bowl sound. The envelope, or in other words, the beat signal, represents the rhythm in the music at which the pitched singing bowl sound changes slowly with time. Figure 3d is the frequency spectrum of the beat rhythm plotted in the frequency range of 0~50 Hz, used in clinical brain waves. As shown in Figure 3d, the strongest beat was observed to occur at 6.68 Hz, while a pair of minor beats appeared at the either side from about 1 Hz to 15 Hz. Note that the frequency of the strongest beat is located in the theta wave band (4~8 Hz), well observed in meditation. Figure 3e is the time–frequency representation of the beat signal, showing the temporal variations in the multiple beat frequencies. The spectrogram was calculated using a short time FFT with a window length of 4 s and a time resolution of 0.5 s. As expected, it is clearly seen that the strongest beat is shown at 6.68 Hz. Its loudness was at a maximum at the beginning of playing the singing bowl (t = 0) and started to decrease rapidly from 10 s to 30 s. Minor multiple beats are seen at the frequencies near 10 Hz, 13.3 Hz, 16.2 Hz and 36 Hz, disappearing within 10~20 s.

### 3.2. Synchronized Activation of Brain Waves at the Beat Frequency

Seven spectral bands were considered in the study, including the five clinical frequency bands (delta: 0~4 Hz, theta: 4~8 Hz, alpha: 8~13 Hz, beta: 13~30 Hz and gamma: 30~50 Hz), the entire frequency range (0~50 Hz) and the beat frequency (6.68 Hz). The mean and standard error of the spectral magnitude of the brain waves recorded for the 17 individuals are analyzed at the temporal middle of each 50 sec singing bowl sound (t = 25, 75, 125, 175, 225, 275, 325 and 375 s). The initial monitoring time, t_i_ = 25 s, represents the temporal middle of the 50 s with no sound, and the final time, t_f_ = 375 s, is that after the last (sixth) singing bowl sound. The spectral magnitude of the brain waves measured in F4 was observed to be similar or slightly larger than those measured in F3. However, there was no statistically significant difference observed between the measurement locations (F3 and F4) in all frequency bands. The ranges of the minimum (*p* = 0.075) to the maximum (*p* = 0.973) *p* values are shown to be large enough to state that the location effects may not be significant. Data collected at each monitoring time were checked for statistical normality using the Shapiro–Wilk test.

The spectral magnitudes of each frequency band of brain waves are different to one another in their initial value. This makes it difficult to compare their temporal changes to one another. To remove the effect of the initial value difference, the magnitude of each frequency band needs to be normalized to the initial value. In addition, the magnitude of the measured brain waves varies from subject to subject. The spectral power of a particular clinical frequency band is often expressed as a ratio (in %) to the total power of the overall frequency range (0~50 Hz) to compensate for the differences in participants.

**Figure 4:**
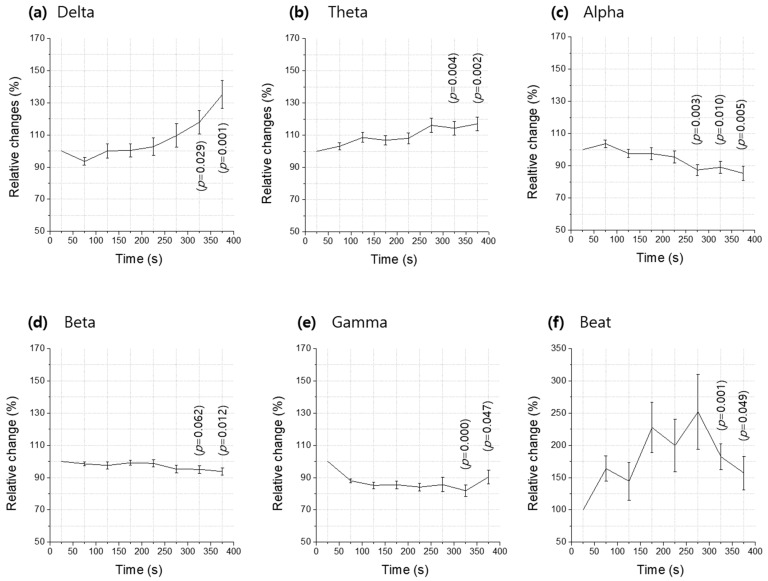
Temporal variations (in %) of each spectral band brain wave magnitude relative to its initial value and normalized to that of the overall frequency band, averaged with data measured at the two positions (F3 and F4) from the participants (*n* = 17) who heard the strongly beating singing bowl sounds repeated six times every 50 s for t = 50~350 s, plotted at every 50 s: (**a**) delta (0~4 Hz), (**b**) theta (4~8 Hz), (**c**) alpha (8~13 Hz), (**d**) beta (13~30 Hz), (**e**) gamma (30~50 Hz) and (**f**) beat (6.68 Hz). Note that the ranges of *p* values are presented for the statistical test on the changes from initial states, and the error bars represent the standard errors.

**Figure A1:**
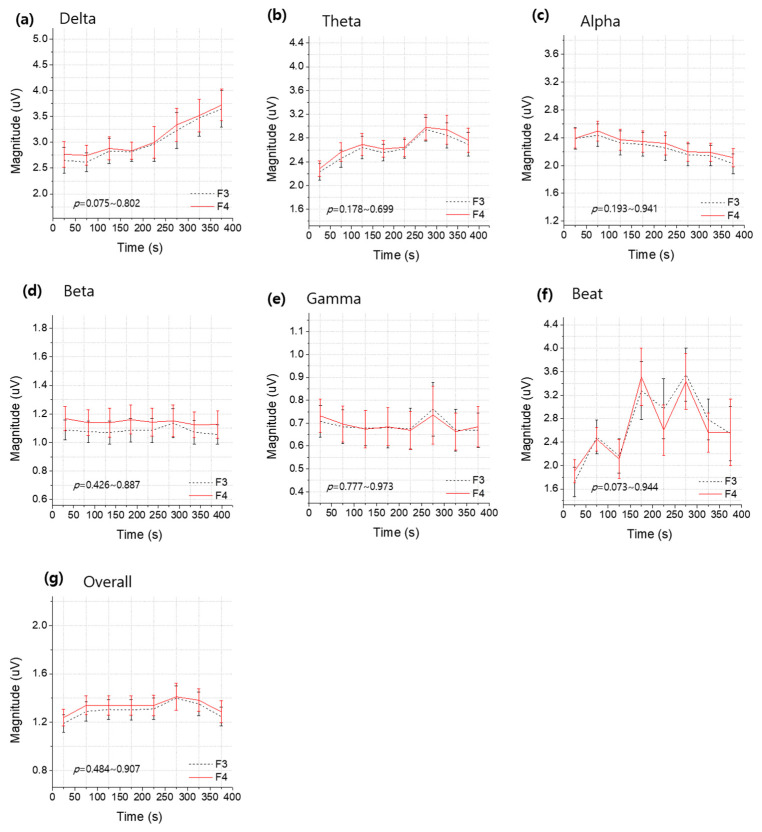
Temporal variations in the magnitudes of each spectral band of the EEG signals measured at F3 and F4 of the 17 participants plotted at every 50 s: (**a**) delta (0~4 Hz), (**b**) theta (4~8 Hz), (**c**) alpha (8~13 Hz), (**d**) beta (13~30 Hz), (**e**) gamma (30~50 Hz), (**f**) beat (6.68 Hz), and (**g**) overall (0~50 Hz). Note that the ranges of *p* values are presented for the statistical test between the measurement locations (F3 and F4), and the error bars represent the standard errors.

Figure 4 shows the temporal changes in the spectral power of the measured brain waves, plotted every 50 s for which the singing bowl was repeatedly played. The mean and standard error of the spectral magnitude of the brain waves recorded for the 17 individuals were analyzed at the temporal middle of each 50 sec singing bowl sound (t = 25, 75, 125, 175, 225, 275, 325 and 375 s). The initial monitoring time, ti = 25 s, represents the temporal middle of the 50 s with no sound before, and the final time, tf = 375 s, is that after the last (sixth) singing bowl sound. In order to more effectively compare the temporal changes in the magnitude of each frequency band of the brain wave, we averaged the values measured from the two locations of F3 and F4. This unification was justified by the statistical finding that the spectral magnitudes of every frequency band were not different between the two locations for the entire experimental duration, as the maximum and the minimum *p* values show in Appendix A Figure A1.

A new parameter of the spectral magnitude of brain waves was introduced to effectively remove the effects of not only the initial value difference but also the subject dependence. Let M(fb, t) be the spectral magnitude of a frequency band of the brain wave at time t. The new parameter A(fb, t), introduced in the present study and defined in Equation (1), is the magnitude of a frequency band of the brain wave normalized to its initial value and to the magnitude of the overall frequency range.

(1) $$A\left(\mathrm{fb},t\right)\left(\mathrm{in}\;\%\right)=\frac{<M\left(\mathrm{fb},t\right)/M\left(\mathrm{fb},\mathrm{ti}\right)>}{<M\left(\mathrm{overall},t\right)/(M\left(\mathrm{overall},\mathrm{ti}\right)>}\;*\;100$$

where fb represents the frequency band, t is the time variable and ti stands for the initial time, which is 25 s in the present study, as illustrated in Figure 4. The numerator of the right-hand side of Equation (1) represents the temporal history of the magnitude of each frequency band relative to its initial value, while the denominator is the temporal magnitude of the overall frequency band relative to its initial value. A(fb, t) stands for the rate of change in the spectral magnitude of each frequency band normalized to that of the whole frequency range (0~50 Hz).

Figure 4 shows A(fb, t) in %, i.e., the rate of change in the spectral magnitude of each frequency band ((a) delta: 0~4 Hz, (b) theta: 4~8 Hz, (c) alpha: 8~13 Hz, (d) beta: 13~30 Hz, (e) gamma: 30~50 Hz, (f) beat: 6.68 Hz), normalized to that of the whole frequency range (0~50 Hz) and averaged with the data measured at the two locations of F3 and F4 for the 17 participants. The temporal changes were plotted at every 50 s for the time from ti = 25 s to tf = 375 s, and the error bar represents the standard error. The data are provided in Table 1, together with the *p* values resulting from the statistical test on each temporal change from the initial value at t = ti. The *p* value (at t = tf) after the experiment is presented Figure 4, and, if it is not the minimum value, the minimum is also provided at its time location.

As expected, the rate of change increased the most at the beat frequency with time (Figure 4f). Among the clinical frequency bands, the increase rate was the largest in the delta wave (135.18%, *p* = 0.001), followed by the theta wave (117.07%, *p* = 0.002). In those two waves located in the low frequency range, the rate of change in the spectral magnitude increased with time, whereas they decreased with time in the high frequency range including alpha, beta and gamma waves. The tendency of the changes maintained during the silent time after the last singing bowl sound, except for the gamma wave and the beat frequency. This trend implies that the largest changes were observed after the last singing bowl sound rather than when the participants heard the last singing bowl sound. This is why the *p* value was at a minimum at t = 375 s rather than at t = 325 s (Figure 4a–d). At the beat frequency, however, the largest increase in the spectral magnitude was observed at the time when the participants heard the fifth singing bowl sound, just before the final one. This can be understood as an extension of the preceding repeated pattern of the (large and rapid) jump and (small and slow) fall, and it is expected to have a spectral magnitude larger than the previous maximum if the participants hear an additional (seventh) singing bowl sound after the last one.

**Figure 5:**
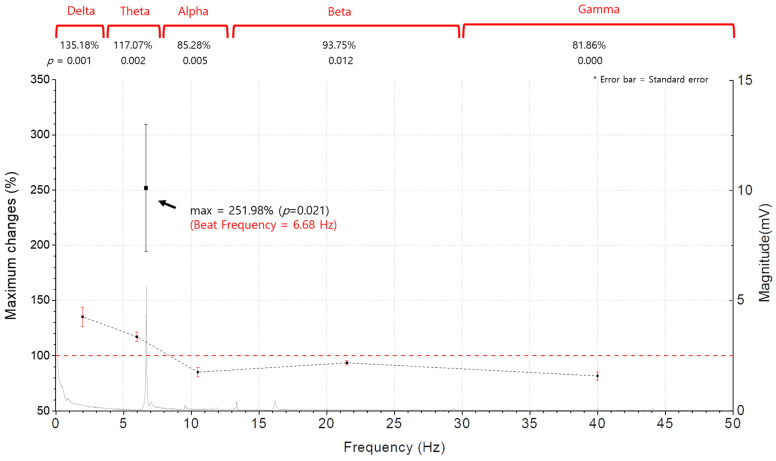
Comparison of the maximum rate of the relative change in spectral magnitude of each spectral band (A(fb,t) in %), together with the frequency spectrum of the beat of the singing bowl sound. The rate of increase is predominant at the beat frequency, which reaches 251.98% (*p* = 0.021) of its initial value at the time (t = 275 s) approaching the end of the experiment. This implies that the brain waves are most effectively synchronized at the beat frequency and activated by the singing bowl sound. Among the five clinical EEG frequency bands, the delta wave increased the most to 135.18% (*p* = 0.001) of its initial state, followed by the theta wave, with a rise of 117.07% (*p* = 0.002). In contrast, the other three spectral bands decreased after the experiment. The gamma wave was down to 81.86% (*p* = 0.000), the alpha wave was down to 85.28% (*p* = 0.005) and the beta wave was down to 93.75% (*p* = 0.012) of its initial state.

Figure 5 compares the maximum rate of the relative changes in the spectral magnitude of each spectral band (A(fb,t) in %), together with the frequency spectrum of the beat of the singing bowl sound. The rate of the increase is predominant at the beat frequency, which reaches 251.98% (*p* = 0.021) of its initial value at the time (t = 275 s) approaching the end of the experiment. This implies that the brain waves are most effectively synchronized at the beat frequency and activated by the singing bowl sound. Among the five clinical EEG frequency bands, the delta wave increased the most to 135.18% (*p* = 0.001) of its initial state, followed by the theta wave with a rise of 117.07% (*p* = 0.002). In contrast, the other three spectral bands decreased after the experiment. The gamma wave was down to 81.86% (*p* = 0.000), the alpha wave was down to 85.28% (*p* = 0.005) and the beta wave was down to 93.75% (*p* = 0.012) of their initial states.

## 4. Discussion

The singing bowl used in this study produces a sound that lasts for more than 50 s after playing it once and has a strong beat at the frequency of 6.68 Hz. When the participants were listening to the singing bowl sound, the spectral magnitudes of their brain waves were shown to increase with time at low frequencies (≤8 Hz, delta and theta waves), whereas they decreased with time at high frequencies (>8 Hz, alpha, beta and gamma waves) (Figure 4). Among the five clinical spectral bands, the rate of increase was the highest for the delta wave (135.18%, *p* = 0.001), followed by the theta wave (117.07%, *p* = 0.002). Under the present experimental conditions, where the participants heard six repeating singing bowl sounds for 300 s, the largest rate of increase (251.98%, *p* = 0.021) was observed at the beat frequency of the singing bowl sound (Table 1). This result suggests that, when the participants were listening to the singing bowl sound, their brain waves were activated and effectively synchronized at the beat frequency.

The beat frequency of the singing bowl sound used in this study belongs to the theta wave spectral band. Numerous studies have observed psychophysiological changes due to the effects of meditation as an increase in theta waves [5,6,7,8,9,15,16,17,18,19,20,21,22]. The present finding that the brain wave is synchronized and activated at the beat frequency located in the theta wave may serve as an academic basis that a singing bowl sound can be used in meditation. In future studies, it would be of interest to consider beat frequencies located in the other clinical spectral bands.

In the present study, in order to observe the response of brain waves to the singing bowl sound, the temporal changes in EEG signals relative to those of the initial resting state were observed. The normalized parameter A(fb,t) defined by Equation (1) is expected to effectively remove subject-dependent effects. A conventional approach of comparing the experimental group to the control may be unnecessary or inappropriate to study the individual response to singing bowl sounds. This study monitors brain waves for the limited time of 400 s from 50 s before the first singing bowl sound to 50 s after the last (sixth) singing bowl sound. As shown in Figure 4a–d, the changes in brain waves were extended even in silence after the last singing bowl sound. As discussed in Section 3.2 regarding the brain wave activity at the beat frequency (Figure 4f), it is interesting to test what would happen if the participants heard an additional (seventh) singing bowl sound. It is of interest to see if the pattern of (large and rapid) jumps and (small and slow) falls would repeat and if the spectral magnitude would increase compared to the present maximum value observed at the fifth singing bowl sound. A future study is suggested to include temporal information when the maximum rate of the increase in brain waves is achieved.

In previous studies on meditation and brain waves, delta waves were observed to increase in the prefrontal cortex [23], as measured at the same location used in the present study. Tei et al. (2009) compared the activity of delta waves using low-resolution electromagnetic tomography (LORETA), where people either meditated (Qigong) or just rested with their eyes closed (control group) [24]. In the frontal lobe of the subjects who meditated, the delta waves were significantly different and stronger than those of the control group. In the present study, delta waves were shown to slightly decrease immediately after the first singing bowl sound, followed by a continuous increase to the highest increase rate (135.18%) among the five clinical brain waves. It is of interest to note that, even after the experiment ended up, the delta waves continued to increase at an enhanced rate. The participants laid down on a bed-chair and had their eyes closed, listening to the singing bowl sound. Such relaxed conditions may easily make the participants feel sleepy and five minutes would be sufficient for some of the participants to fall asleep. In fact, a few of them were found to snore in their sleep. Once they went into stage 1 sleep, delta waves were expected to keep increasing with time. Even after listening to the last singing bowl sound for 50 s, the participants kept laying down on the bed chair and, with no singing bowl sounds, additional EEG signals were measured for 50 s, which were expected to contain more delta waves.

A reduction in alpha waves is known to be a common phenomenon in the entire range of relaxation therapy [25]. Numerous prior studies have reported a decrease in the alpha waves in yoga or transcendental meditation [7,8,18,21,26,27]. Various studies have also reported a decrease in alpha waves by approximately 50% due to an increase in theta waves in the first stage of sleep [25,28]. In the present study, the spectral magnitudes of alpha waves were smaller than those at rest before the experiment, and decreased steadily as the participants started to hear the singing bowl sound, reaching 85.28% of the initial states at the end of the experiment. The observed continuous decrease with time in alpha waves is attributed to the effect of the singing bowl sound that may induce the participants to relax or meditate.

It appears that beta waves did not change over time with significance (Figure 4 and Figure 5), but they were found to decrease by 6.25% (*p* = 0.012) at the end of the experiment. A number of studies have shown a decrease in beta waves during meditation [29,30,31]. In particular, beta wave decreases were reported to be associated with a relaxation response or Zen meditation [29,30,31]. A meditation process was not considered in the present experiment, but the participants lay down on a chair-bed and were listening to the singing bowl sound. The observed decrease in beta waves is speculated to result from the relaxed resting states into which the participants gradually slipped during the experimental period.

The gamma wave activity during meditation is controversial. Some studies have shown a decrease in gamma waves during meditation [23], while the other studies reported an increase in gamma waves [32,33,34]. The present study shows that the gamma waves continuously decreased by up to about 12~18% when the participants were listening to the singing bowl sound. However, gamma waves were observed to rise again, approaching the initial state, as the participants stopped hearing the sound. It should be noted that the present study employed a singing bowl sound whose beat frequency is located in the theta wave region and, in future studies, it would be of interest to look at the gamma wave response to a singing bowl sound whose beat frequency is located in the gamma wave region.

The present study was based on brain waves measured at limited locations (F3 and F4). The measurement locations of F3 and F4 are known to be sensitive to brain activity during meditation [5,8,10,12,13,14,35,36,37], and they are reasonable locations for the singing bowl meditation of the present study. Further studies with measurements at various positions are required to expand and generalize the observed synchronized activation of the brain waves. In addition, the present results were obtained from a relatively small number of participants (*n* = 17). Fortunately, Shapiro–Wilk tests confirmed the normal distributions of the measured data, which ensured the reliability of the statistical tests performed in the present study.

The beat of the singing bowl sound is determined by the size, material and structure of the instrument. The various singing bowls used in meditation are classified in accordance with the fundamental frequencies of their sounds as musical key tones. The fundamental frequency of the singing bowl used in the present experiment was approximately 480 Hz, which musically corresponds to B4. As shown in Figure 3c, the singing bowl sound used in this study was composed of multiple harmonic components at 773.15 (G5), 1102.56 (C#6), 1464.81 (F#6) and 1870.86 Hz (A#6). The tonal property of the singing bowl is not affected by the manner of playing and the sound volume. Nevertheless, the sound intensity and tonal properties are important psycho-acoustical parameters [38] which are expected to affect the brain waves independently. In the present experiment, an arbitrary single singing bowl was chosen, and the playing method and the sound intensity were not precisely controlled. A follow-up study is suggested to explore the interesting aspects of how the synchronized activation of the brain waves is related to playing techniques and the intensity of the beating sound for singing bowls with various key tones.

## 5. Conclusions

The beat frequency of the singing bowl sound used in this study was determined to belong to the theta wave region, which is known to increase during meditation. In this experiment, the brain waves of the participants who heard the singing bowl sound were observed to be activated in a few minutes with its strong beat rhythm. This study presents experimental evidence that the singing bowl sound likely activates brain waves that are effectively synchronized with the beating rhythm. The present findings underpin that the strongly beating singing bowl sound facilitates meditation, relaxation and psychological stability.

## Figures and Tables

**Table 1:** Temporal variations (in %) in the spectral band brain wave magnitudes relative to their initial values (0~50 s), normalized to those of the overall frequency band and averaged the data measured at the two positions (F3, F4) of the participants (*n* = 17) who heard the strongly beating singing bowl sounds repeated six times at every 50 s for t = 50~350 s. (†: maximum change).

Time (s)		Delta Wave		Theta Wave		Beat Frequency		Alpha Wave		Beta Wave		Gamma Wave	
		Changes in EEG (%)	*p*	Changes in EEG (%)	*p*	Changes in EEG (%)	*p*	Changes in EEG (%)	*p*	Changes in EEG (%)	*p*	Changes in EEG (%)	*p*
Before exp.	0~50	100	-	100	-	100	-	100	-	100	-	100	-
Experiment	50~100	93.52	0.014	103.16	0.176	163.90	0.006	103.69	0.137	98.48	0.248	87.99	0.000
	100~150	100.09	0.985	108.57	0.015	144.40	0.160	97.69	0.411	97.54	0.280	85.18	0.000
	150~200	100.37	0.933	106.85	0.037	227.66	0.006	97.57	0.533	99.16	0.579	85.53	0.000
	200~250	102.71	0.641	108.09	0.031	199.67	0.032	95.47	0.275	98.78	0.586	84.10	0.000
	250~300	109.62	0.220	116.16	0.003	251.98 ^†^	0.021	87.40	0.003	95.19	0.067	85.65	0.006
	300~350	117.95	0.029	114.34	0.004	182.19	0.001	89.07	0.010	95.09	0.062	81.86 ^†^	0.000
After exp.	350~400	135.18 ^†^	0.001	117.07 ^†^	0.002	157.06	0.049	85.28 ^†^	0.005	93.75 ^†^	0.012	90.41	0.047

## Data Availability

Not applicable.
